# Assessing disaster-smart school governance to advance an integrated evaluation framework

**DOI:** 10.4102/jamba.v18i1.2101

**Published:** 2026-09-25

**Authors:** Roni Ekha Putera, Aqil Teguh Fathani, Zikri Alhadi, Ramadhan Pancasilawan

**Affiliations:** 1 Department of Public Administration, Faculty of Social and Political Science, Universitas Andalas, Padang, Indonesia; 2 Doctoral Program of Policy Studies, Faculty of Social and Political Sciences, Universitas Andalas, Padang, Indonesia; 3 Department of Political Science, Faculty of Social and Political Science, Universitas Pembangunan Nasional Veteran, Jakarta, Indonesia; 4 Department of Public Administration and Research Centre for Policy, Governance, Development and Empowerment, Faculty of Social Sciences, Universitas Negeri Padang, Padang, Indonesia; 5 Department of Public Administration, Faculty of Social and Political Sciences, Universitas Padjajaran, Bandung, Indonesia

**Keywords:** disaster-smart school, governance, disaster risk reduction, institutional capacity, monitoring and evaluation, integrated evaluation framework, school safety policy

## Abstract

This research examined the governance factors influencing disaster-smart school governance (DSSG) and suggests a comprehensive evaluation framework to enhance resilience within educational systems. Despite global commitments outlined in the Sendai Framework and the comprehensive school safety framework, governance outcomes in school-based disaster risk reduction (DRR) remain inconsistent, primarily due to fragmented institutional capacity and a lack of empirical clarity regarding the significance of various governance dimensions. The research employed a quantitative design using partial least squares-structural equation modelling (PLS-SEM) to analyse data from disaster-smart schools, focusing on the impact of five governance constructs. The results show that risk knowledge (X2) has the strongest positive association with DSSG (*β* = 0.472, *p* < 0.001, F^2^ = 0.289), followed by learning, monitoring and accountability (X5) (*β* = 0.277, *p* < 0.001, F^2^ = 0.184). Leadership and strategic commitment (X1) and stakeholder participation (X3) show smaller but significant associations, whereas operational preparedness and/or resource management (X4) is not statistically significant (*β* = 0.017, *p* = 0.685, F^2^ = 0.001). These findings contradict prevailing assumptions that emphasise leadership and participatory engagement as the main factors influencing safety governance. The importance of system-centred governance is based on institutional strength, regulatory coherence, resource distribution and ongoing learning.

**Contribution:**

The research presents an evidence-based evaluation framework, highlighting that disaster-smart schools emerge from continuous, measurable and systematic governance processes.

## Introduction

The effectiveness of disaster management systems is continually challenged by a variety of hazards and ongoing governance shortcomings, shaped by the fragmented roles of stakeholders (Forino, Von Meding & Brewer 2015; Zweig 2025). Cassedy and Faulkner asserted that regions susceptible to disasters, characterised by stagnant institutional learning, often encounter complex managerial challenges (Cassedy 1991; Faulkner 2001). Hence, disaster governance poses a challenge due to the necessity for ongoing cognitive reflection, swift and coordinated decision-making and adaptive responses in contexts marked by significant uncertainty.

Issues of centralisation and the intricate challenges associated with decentralisation continue to entangle the discourse on disaster governance. Tommasi and Weinschelbaum (2007) support centralisation and posit that a cohesive command structure enhances crisis response effectiveness. Also, Nowell et al. (2018) argue that centralised systems often fail due to communication asymmetry, vertical fragmentation and restricted regional autonomy, resulting in bifurcated governance that disconnects decision-making from on-the-ground realities (Bouraoui & Lizarralde 2013; eds. Wisner, Gaillard & Kelman 2012).

Empirical evidence suggests that hierarchical and technocratic governance structures in developing countries, such as Indonesia, often lead to inflexible coordination mechanisms that hinder local problem-solving capabilities (Izadkhah & Hosseini 2005; Smoke 2015).

Consequently, frontline operators or individuals familiar with conditions in disaster zones are frequently overlooked due to their limited authority in making rapid decisions, leading to inadequate responses.

The archipelagic geography and tectonic position of Indonesia intensify governance challenges, rendering the nation susceptible to earthquakes, tsunamis, volcanic eruptions, floods and landslides (BNPB 2023). The education sector occupies a dual strategic role in this high-risk environment, being both susceptible to disasters and essential for promoting early disaster awareness and preparedness. Disaster-smart school governance (DSSG) has emerged as a vital strategy for effective and sustainable disaster risk reduction (DRR).

The United Nations Office for Disaster Risk Reduction (UNDRR) emphasises three pillars of school safety through the Comprehensive School Safety Framework (CSSF): resilient educational facilities, adequate school-based disaster risk management and the incorporation of disaster education into curricula (UNDRR 2022). This framework aims to enhance institutional capacity while necessitating innovative policies that can effectively address the complex relationships among local, national and global DRR challenges. The Sendai Framework for Disaster Risk Reduction 2015–2030 identifies resilience building and institutional reconstruction as fundamental components of effective governance for DRR (Aitsi-Selmi et al. 2016; Kelman 2015). Sectoral fragmentation, incomplete implementation and a lack of institutional innovation impede Indonesia’s disaster governance system.

At the school level, governance limitations lead to inconsistent DRR initiatives, unsustainable programming and a significant dependence on external actors, including non-governmental organisations (NGOs) and short-term donor projects (Izadkhah & Hosseini 2005). Weak institutional capacity, inadequate disaster literacy among educators and students and limited stakeholder engagement hinder the implementation of DSSG (Bansal, Mukherjee & Gairola 2017; Luetz 2020). With the intensification of climate-related disasters, educational institutions must evolve from being passive recipients of protection to becoming proactive agents of community resilience (Gaillard & Mercer 2013). This change needs systematic, evidence-based and participatory strategies to make the education sector more resilient (Elangovan & Kasi 2015; Shaw, Shiwaku & Takeuchi 2016).

Disaster-smart school governance is increasingly acknowledged as an essential governance approach (Mowen 2015; Tipler et al. 2017); however, Indonesia currently lacks a unified and innovative governance framework that incorporates institutional, behavioural and participatory elements into a school-based DRR system. Schools function as educational settings and protective entities, stabilising communities in times of disaster. Disaster-smart school governance represents an adaptive and collaborative governance model that incorporates infrastructure resilience, stakeholder engagement, a disaster-smart culture and risk-based management practices.

Existing research also criticises the patchy implementation of DSSG; many projects fail due to confusing conceptual frameworks, insufficient cross-sectoral coordination and ineffective policy design (Amri et al. 2022). Overlapping duties, misaligned human resources and insufficient integration of local capacities and available resources all lead to recurring governance failures in constructing disaster-resilient schools (Purwanto et al. 2024). These issues underscore the pressing need for policy formulations that transcend normative constraints and prioritise innovation, interdisciplinary collaboration and robust evidence-based approaches.

Disaster-smart school governance necessitates more than mere awareness campaigns or resilient infrastructure. It demands a governance framework that incorporates institutional structures, stakeholder engagement, behavioural competencies, curriculum integration and adaptive risk management. There is a necessity for innovative policies to transition school governance from a reactive to an anticipatory model, establishing school communities as hubs for disaster education, early warning communication and collective resilience.

Effective policy design depends on conceptual clarity, operational feasibility, stakeholder synergy and the assurance of long-term sustainability. A systematic assessment is necessary to evaluate the progress, coherence and effectiveness of current DSSG practices. This study employs a quantitative approach to identify best practices, diagnose governance gaps and develop a participatory, evidence-based policy framework aimed at enhancing DSSG. The primary question of this research is to what extent the DSSG assessment system facilitates systemic, integrated and sustainable disaster risk management.

This inquiry corresponds with the assertion that DSSG policies necessitate the reorganisation of governance frameworks to facilitate multi-actor collaboration, curriculum integration and community engagement. An appropriate policy design enables schools to function as both educational institutions and community learning centres, enhancing disaster awareness, promoting collective preparedness and reinforcing local resilience.

## Literature review

### Disaster management theory and practice

Disaster management is a multidisciplinary and multilevel process designed to mitigate the impact of disasters through preparedness, response and recovery strategies. Disaster management involves intricate planning, coordination and governance frameworks that span various levels of government and community stakeholders (Fathani, Putera & Mohamed 2024; Ryan et al. 2020). The Sendai Framework for Disaster Risk Reduction 2015–2030 articulates a perspective that emphasises the importance of robust disaster risk governance, improved understanding of risks and enhancement of resilience among individuals and essential infrastructure. This framework promotes the incorporation of DRR into national and local development plans, especially within the education sector, by identifying schools as essential locations for risk governance and preparedness (Pearson & Pelling 2015).

In this study, DSSG refers to an integrated school governance approach that combines risk knowledge, leadership, stakeholder participation, operational preparedness, monitoring, accountability and institutional learning. The term ‘school resilience’ is used to describe the broader capacity of a school to anticipate, absorb, adapt to and recover from disaster risks, while ‘institutional capacity’ refers to the organisational resources, procedures and accountability mechanisms that enable DSSG implementation.

Conventional frameworks of the disaster management cycle delineate four interconnected phases – mitigation, preparedness, response and recovery – as essential elements of thorough emergency management (Carter 2008). Recent studies challenge the overly linear conception of this cycle, which overly defines the phases and minimises the significance of feedback loops.

Systematic assessments indicate that effective disaster management depends on the integration of tasks within organisational frameworks, decision-making processes and cross-sector coordination, highlighting governance as a critical factor rather than solely as an operational concern (Khan et al. 2023).

This study situates DSSG within a broader transition from a hazard-focused, response-driven model to a risk-governed framework that prioritises proactive capacity, institutional learning and accountability in the educational context.

### Risk governance and stakeholder participation

The risk governance framework highlights that disasters exhibit complexity, uncertainty and ambiguity, necessitating inclusive decision-making processes alongside technocratic solutions (Pearson & Pelling 2015). Also, Renn (2015) posits that stakeholder and public engagement are essential for compelling and legitimate risk governance, recommending participation formats that are adapted to different levels of complexity and uncertainty. Nowell et al. (2018) assert that governance encompasses self-organising and steering processes within complex societies; it is characterised by adaptability, flexibility and openness to cross-sectoral participation.

These have facilitated the emergence of community-based disaster risk management (CBDRM), emphasising local knowledge, social networks and the collaborative development of risk reduction strategies. Systematic reviews indicate that community engagement enhances early warning uptake, evacuation decisions and preparedness behaviours when integrated into ongoing participatory processes, rather than through isolated awareness campaigns (Ryan et al. 2020). There remains ongoing debate regarding the institutionalisation of participation: whether local actors are genuinely empowered to influence priorities or are invited to execute decisions predetermined at higher levels.

Schools hold a critical role in this discourse. They serve as education providers, community hubs and, in numerous countries, designated evacuation centres. School disaster management occupies a critical position at the convergence of risk governance and educational governance. The concept of DSSG emphasises that schools should not only adhere to externally established DRR procedures, but also create their own internal frameworks for leadership, role clarity, stakeholder engagement (including students, teachers, parents and local government) and ongoing self-assessment.

### Resilience in disaster-responsive school governance

Resilience is a fundamental concept in modern disaster research. Manyena (2006) characterises resilience as the ability of a system – comprising individuals, communities or institutions – to withstand disturbances, adapt and transform while preserving or improving its fundamental functions. This definition critiques limited perspectives of resilience that regard it merely as a return to a pre-disaster condition, emphasising instead the concept of bouncing forward through processes of learning and innovation.

Resilience, viewed through a social-ecological lens, is rooted in intricate interactions among environmental, economic and institutional spheres (Fathani et al. 2024; Folke 2016). In hazardous areas, school resilience encompasses not only the safety of the buildings, but also the ability to operate the school effectively. These include the capacity of school leaders to make prompt decisions amid uncertainty, coordinate with external agencies, manage conflicting demands when schools serve as shelters, and maintain educational continuity through alternative methods.

The literature indicates a tension between employing resilience as a descriptive concept and its translation into operational, measurable dimensions. This tension directly drives the development of assessment tools aimed at capturing the governance aspects of resilience in educational institutions, extending beyond infrastructural safety and basic emergency procedures.

### Assessment tools for disaster-smart school governance

Indonesia, prone to frequent earthquakes, tsunamis, floods and volcanic eruptions, has enhanced its national disaster management system through normative measures. However, empirical evaluations reveal discrepancies between established regulations and their practical implementation. Inan, Beydoun and Pradhan (2018) demonstrate that a formal disaster management cycle is in place, and the results regarding the reduction of loss of life and economic damage are inadequate due to challenges in implementation and insufficient institutional coordination.

In the education sector, various school safety and disaster preparedness programmes are present; however, evidence regarding the extent to which schools have established mature governance capacities – explicit delegation of DRR responsibilities, incorporation of DRR into school development plans and systems for ongoing evaluation – is limited.

This context underscores the importance of assessment tools that effectively address governance quality and institutional arrangements within disaster-prone educational systems, particularly in developing nations. In response to the necessity for systematic evaluation, researchers have created various instruments to assess a school’s capacity to manage disasters.

The School Disaster Resilience Assessment (SDRA) is a prominent tool for assessing the disaster resilience of educational systems. This analysis examines five areas: physical conditions, human resources, institutional issues, external relationships and natural conditions (Shaw et al. 2016).

This emphasises the resources available to schools (such as infrastructure, equipment and documented plans) and their responses during drills or emergencies, rather than the operation of governance processes. Dimensions like leadership commitment and clarity of roles and responsibilities are frequently represented only indirectly or omitted entirely. Frequently, we only document roles and responsibilities, decision-making procedures, accountability mechanisms, stakeholder participation in planning and lessons learned from near-miss events indirectly or not at all. Comparative reviews suggest that numerous tools have been developed within specific hazard or cultural contexts and may require adaptation for broader application in diverse, resource-limited environments.

These indicate a gap in both concept and methodology. Although tools for evaluating school disaster resilience are available, there remains a scarcity of assessment frameworks that are explicitly based on risk governance theory and specifically designed for assessing DSSG. Few tools effectively integrate principles from risk governance, such as inclusiveness, transparency and responsiveness, alongside resilience thinking, which includes learning, adaptability and transformability and educational management. This integration is crucial for developing a comprehensive set of governance indicators that can assess the maturity of disaster management practices at the school level and inform continuous improvement.

### Research gap and hypothesis development

The literature on disaster management suggests that technical capacity alone is insufficient; governance structures, community engagement and institutional learning have a profound impact on outcomes. In the realm of DRR in school, particularly in developing nations like Indonesia, there exists a deficiency of assessment tools that is frame school as proactive governance entities within comprehensive disaster management frameworks, rather than as mere recipients of national directives; evaluate governance quality and institutional maturity, in addition to physical safety or preparedness initiatives; and flexible to various hazard profiles and resource contexts, while maintaining conceptual integrity and measurement reliability.

To address these gaps, it is essential to develop an assessment framework that focuses explicitly on DSSG, converting theoretical principles of risk governance and resilience into practical, context-sensitive indicators. This framework can assist schools, local governments and national policymakers in evaluating governance performance, pinpointing key areas for capacity enhancement and establishing ongoing improvement in education systems vulnerable to disasters.

Based on risk governance, resilience governance and the CSSF, this study derives five governance dimensions that are expected to be associated with DSSG: Leadership and Strategic Commitment (X1), Risk Knowledge (X2), Stakeholder Participation (X3), Operational Preparedness/Resource Management (X4) and Learning, Monitoring and Accountability (X5). These dimensions represent the institutional, cognitive, participatory, operational and evaluative foundations of school-based DRR. Accordingly, the following hypotheses are proposed:

H1: Leadership and Strategic Commitment is positively associated with DSSG.

H2: Risk Knowledge is positively associated with DSSG.

H3: Stakeholder Participation is positively associated with DSSG.

H4: Operational Preparedness/Resource Management is positively associated with DSSG.

H5: Learning, Monitoring and Accountability is positively associated with DSSG.

## Research methods and design

Exploratory quantitative research was employed to analyse the responses of participants in a policy design study aimed at enhancing disaster-savvy school governance in Padang, West Sumatra Province, Indonesia. This design was selected to achieve the study’s primary objective of identifying the relationship between exogenous and endogenous variables, excluding the use of a median factor in the assessment. The relationships among variables were assessed using SmartPLS-based structural equation modelling (SEM), allowing for the evaluation of the proposed hypotheses through statistical and numerical analysis. Table 1 presents the variables.

**TABLE 1:** Research variable and indicator.

Variable	Indicator	Key theoretical
Leadership and strategic commitment (X1)	X1.1 strategic prioritisation of DRR; X1.2 leadership commitment; X1.3 formal DRR committee; X1.4 clear emergency decision-making.	Risk governance; school DRR leadership
Risk knowledge (X2)	X2.1 hazard and vulnerability assessment; X2.2 use of risk data and local knowledge; X2.3 risk communication; X2.4 integration into teaching.	Sendai Framework Priority 1; risk communication
Stakeholder participation (X3)	X3.1 involvement of teachers and staff; X3.2 student participation; X3.3 parent and community engagement; X3.4 external partnerships.	Community-based DRR; participatory governance
Operational preparedness (resource management) (X4)	X4.1 written emergency plans; X4.2 drills and simulations; X4.3 emergency facilities; X4.4 resource allocation; X4.5 continuity of learning	Comprehensive school safety; preparedness
Learning, monitoring and accountability (X5)	X5.1 monitoring and review; X5.2 documentation and lesson-learning; X5.3 integration into school improvement plans; X5.4 accountability and feedback channels.	Governance cycle; organisational learning; accountability
Disaster-smart school governance (Y1)	Y1.1 risk-informed school governance; Y1.2 DRR integration into school planning; Y1.3 institutional coordination for school safety; Y1.4 accountability and evaluation of DRR practices; Y1.5 sustainability of school-based DRR programmes.	Risk governance; comprehensive school safety; resilience governance; school-based DRR

DRR, disaster risk reduction.

The endogenous construct in this study is DSSG (Y), which refers to the extent to which school possess integrated governance capacity to manage disaster risk through risk-informed planning, institutional coordination, preparedness practices, monitoring, accountability and continuous learning. DSSG is treated as a governance outcome that reflects the integration of institutional, participatory, operational and evaluative dimensions in school-based DRR.

Quantitative data were gathered via a questionnaire distributed to 215 respondents to evaluate the direct and comprehensive effects of variables X1, X2, X3, X4 and X5 on the endogenous variable (Y). This study identifies relationships and provides practical contributions to enhancing disaster management through a school governance framework designed to address future disasters. Table 2 presents the details of the respondents.

**TABLE 2:** Respondent profile.

Variable	Category	Frequency (*n*)	%
Total respondents	215	215	100
Total schools	22	22	100
Gender	Male	143	66.51
	Female	72	33.49
Age group (years)	≤ 30	45	20.93
	31–40	63	29.30
	41–50	76	35.34
	> 50	31	14.43
Role at school	Principal and vices	22	10.23
	Teacher	76	35.36
	Administrative staff	54	25.11
	Support staff	63	29.30
Teaching experience (years)	≤ 5	55	25.58
	6–10	64	29.76
	11–15	65	30.23
	> 15	31	14.41
Main hazard exposure	Earthquake	35	16.27
	Flood	10	4.65
	Landslide	22	10.23
	Tsunami-coastal	92	42.79
	Multi-hazard	56	26.06

Respondents were selected from 22 disaster-smart schools in Padang, West Sumatra, using purposive sampling. Eligible respondents included principals, vice-principals, teachers, administrative staff and support staff who were involved in, or familiar with, school governance and DRR practices. The questionnaire was developed from the Sendai Framework, the CSSF and risk governance literature, and was structured using a five-point Likert scale. Prior to data collection, the instrument was reviewed for content relevance and clarity. Participation was voluntary, respondents were informed about the purpose of the study, and responses were treated anonymously and confidentially.

Additionally, data collection employed a Likert-scale questionnaire utilising a 1–5 scale that encompassed five exogenous variables (X1–X5) and one endogenous variable (Y). The distribution of respondents’ answers can indicate the presence of formal preparedness policies and practices in schools, as well as the absence of integrated risk management and sustainability components. The findings establish a significant foundation for subsequent analysis utilising the partial least squares-structural equation modelling (PLS-SEM) approach.

The research procedure commenced with the planning and preparation phase, which involved formulating the problem, objectives and hypotheses. Data collection was conducted via the distribution of questionnaires and interviews. The data were analysed using SmartPLS to investigate the relationships among the variables. Structural equation modelling was chosen for its capacity to concurrently examine relationships among variables, accounting for both direct and indirect effects. Testing occurred in two phases: firstly, a measurement model assessed construct validity and reliability; secondly, a structural model analysed the relationships among the hypothesised constructs within the research framework.

In accordance with the guidelines set forth by Hair et al. (2014), structural modelling analysis was performed through three testing steps: hypothesis testing, evaluation of the outer model and assessment of the inner model. The evaluation of statistical power utilised a Cronbach’s alpha of 0.6, a composite reliability of 0.70, and an average variance extracted (AVE) exceeding 0.50. Furthermore, a *p*-value threshold of 0.05 was employed to establish significance, as outlined by Chin and Scheines (Chin 1998; Scheines, Hoijtink & Boomsma 1999). This method assessed the relationships among various variables by calculating regression paths; also, Bandalos (2002) and Sardeshmukh and Vandenberg (2017) employed a threshold to calculate effect sizes, specifically *F*^2^ = 0.150.

The evaluation of the deep or structural model included a multicollinearity test, yielding a variance inflation factor (VIF) value of less than 5. Conversely, a value greater than 5 indicates the presence of multicollinearity among constructs, necessitating the recalculation of research indicators. The results incorporated path coefficients, *p*-values, upper and lower bounds, effect size (*F*^2^), coefficient of determination (*R*^2^), *Q*^2^ and goodness-of-fit (GoF).

Iterative processing was employed to assess the research variables through two methods of manipulation: calculating values based on indicators and latent variables and explicitly evaluating relationships between latent variables through external estimates. The significance of variables was evaluated through the PLS Bootstrap method; endogenous variables are affected by a combination of exogenous variables derived from the observed elements, specifically X1–X5.

### Ethical considerations

Ethical clearance to conduct this study was obtained from the Universitas Andalas Research Ethics Committee (Ref. No. B/468/UN16.08.D/TP.02.02/2025).

## Results

### Outer model results

The study comprised 215 participants, including principals, deputy principals, teachers, administrative personnel and school committee administrators. Table 2 presents the proportion of respondents, which closely correlates with the proposed variables and indicators. The respondents’ findings suggest that they are currently in a productive phase and have adequate experience in school governance, particularly in implementing governance and policies for disaster-safe education.

The PLS algorithm assessed the validity and reliability of the research data, demonstrating consistency with the statements and categorising it as a valid instrument that meets the practical measurement requirements of the research variables. The external load, when assessed against the binding indicators, was determined to be valid and acceptable, with a value exceeding 0.60.

The indicators utilised demonstrated the reliability of the variables and the acceptability of the measurement model. The acceptance of indicators suggests the absence of conflicts that could undermine other pertinent coefficients (refer to Table 3).

**TABLE 3:** Outer model results.

Variable	Measurement items	Outer loading	Cronbach’s alpha	Composite reliability	AVE
X1	-	-	0.861	0.881	0.704
	X1.1	0.840	-	-	-
	X1.2	0.882	-	-	-
	X1.3	0.853	-	-	-
	X1.4	0.779	-	-	-
X2	-	-	0.915	0.929	0.797
	X2.1	0.844	-	-	-
	X2.2	0.927	-	-	-
	X2.3	0.930	-	-	-
	X2.4	0.866	-	-	-
X3	-	-	0.813	0.983	0.594
	X3.1	0.809	-	-	-
	X3.2	0.848	-	-	-
	X3.3	0.671	-	-	-
	X3.4	0.744	-	-	-
X4	-	-	0.866	0.871	0.717
	X4.1	0.890	-	-	-
	X4.2	0.908	-	-	-
	X4.3	0.887	-	-	-
	X4.4	0.683	-	-	-
X5	-	-	0.863	0.935	0.713
	X5.1	0.902	-	-	-
	X5.2	0.846	-	-	-
	X5.3	0.890	-	-	-
	X5.4	0.728	-	-	-

AVE, average variance extracted.

Indicators X1.5 and X4.5 were removed because their outer loading values did not meet the acceptable measurement threshold and weakened the overall measurement quality of their respective constructs. The removal was conducted to improve indicator reliability, convergent validity and the parsimony of the measurement model, while retaining the conceptual integrity of leadership and strategic commitment (X1) and operational preparedness/resource management (X4). The results of the convergent validity test indicated that nearly all indicators exhibited loading factor values exceeding 0.60, specifically ranging from 0.671 to 0.935. Additionally, the AVE values for each variable fell within the range of 0.570 to 0.832, thereby satisfying the established measurement threshold.

Additionally, internal reliability was assessed through Cronbach’s alpha and composite reliability (CR), with a minimum threshold of 0.70. The results for all items met the threshold, with Cronbach’s alpha values ranging from 0.861 to 0.935 and composite reliability values ranging from 0.853 to 0.940. The research instrument is deemed reliable, confirming that the indicators employed effectively elucidate the latent variables.

To provide a more complete reporting of the PLS-SEM measurement model, convergent validity, internal consistency reliability and discriminant validity were assessed before interpreting the structural model. Convergent validity was confirmed through outer loading values above the acceptable threshold and AVE values exceeding 0.50. Internal consistency reliability was supported by Cronbach’s alpha and composite reliability values above the recommended threshold. Discriminant validity was also examined to ensure that each latent construct was empirically distinct from the others.

Each dimension possesses distinct criteria and rationale for mutually reinforcing one another within the research instrument. Each indicator employed exerts a substantial influence on the measurement. The indicators for variable X1 exhibit a value range of 0.779 to 0.882, with indicator X1.2 being the predominant one, as it effectively elucidates and represents the commitment of the dominant leadership. Variable X2, ranging from 0.844 to 0.930, is primarily influenced by indicator X2.3, which highlights the importance of two-way communication and rejects hierarchical or top-down methodologies.

Variable X3, exhibiting a value range of 0.671 to 0.848, is primarily influenced by indicator X3.2, which underscores the necessity of full participation from all students in achieving effective disaster management outcomes. Variable X4, exhibiting a value range of 0.683, is predominantly influenced by indicator X4.2, which highlights the routine simulation practices implemented by the local government and mandates participation from all students. Variable X5, which ranges from 0.728 to 0.902, is primarily influenced by indicator X5.1, highlighting the importance of routine evaluation and monitoring of all policies approved by stakeholders. The external content demonstrates a mutually reinforcing correlation, indicating that risk enhancement is essential for developing a DSSG assessment.

### Inner model results

In PLS-SEM, the inner model in direct effect analysis indicates the quality, accuracy, strength and feasibility of the structural model that delineates the relationships among latent constructs. Consequently, it necessitates a set of stringent statistical and substantive evaluation criteria. The fulfilment of the VIF, *R*^2^, *p*-values for significance of path coefficients, *F*^2^, *Q*^2^ and standardised root mean square residual criteria collectively underpins the argumentation of the estimated inner model, demonstrating its adequate explanatory, predictive and stability capabilities to substantiate the theoretical findings and empirical implications of the research.

The structural model should be free from collinearity issues by ensuring that the VIF values for the exogenous constructs remain below the recommended threshold of 5. These ensure that the path coefficient estimates are accurate and that collinearity does not compromise these estimates (Hair et al. 2019). The findings indicated that all exogenous constructs displayed VIF values below 5, with a range from 1.339 to 4.637. These suggest the absence of multicollinearity issues, thereby confirming the reliability of the regression coefficient estimates.

The structural model was evaluated through collinearity assessment, path coefficient significance, explanatory power, effect size and predictive relevance. Collinearity was assessed using VIF, while the significance of structural paths was examined through bootstrapping. *R*^2^ was used to assess explanatory power, *F*^2^ to evaluate the relative contribution of each exogenous construct, and *Q*^2^ to examine predictive relevance. These procedures were included to provide a more transparent reporting of the PLS-SEM analysis.

The relationships between constructs were evaluated using a bootstrapping procedure, focusing on the *t*-statistic and *p*-value of the path coefficients. The relationships between constructs were evaluated using a bootstrapping procedure, focusing on the *t*-statistic and *p*-value of the path coefficients. Statistically significant and directional coefficients provide empirical support for the hypothesised structural relationships within the research model (Hair et al. 2019; Henseler & Sarstedt 2013). However, given the cross-sectional nature of the data, the results are interpreted as evidence of association among constructs rather than as definitive structural association. The constructs’ strength was evaluated using the coefficient of determination (*R*^2^), categorised as substantial (*R*^2^ > 0.75), moderate (0.50 < *R*^2^ < 0.75) and weak (*R*^2^ < 0.25) (Hair et al. 2017; Sarstedt et al. 2019). The *F*^2^ effect size was employed to evaluate the contribution of each exogenous construct to the endogenous construct in the direct structural relationship (Cohen 1988; Hair et al. 2019). Refer to Table 4 for the results of the structural evaluation.

**TABLE 4:** Inner model results.

Variable	Original sample (O)	Sample mean (M)	s.d.	*t* statistics (|O/s.d.|)	Path coefficient Confidence interval (95%)		*p*	*F*-Square
					2.5%	97.5%		
X1 > Y	0.172	0.168	0.052	3.303	0.062	0.270	0.001	0.061
X2 > Y	0.472	0.469	0.064	7.319	0.342	0.598	0.000	0.289
X3 > Y	0.115	0.118	0.033	3.537	0.051	0.180	0.000	0.056
X4 > Y	0.017	0.014	0.041	0.406	−0.070	0.091	0.685	0.001
X5 > Y	0.277	0.285	0.056	4.912	0.178	0.398	0.000	0.184

s.d., standard deviation.

The findings indicated that X1 exerted a positive effect of 17.2% (0.172), which was statistically significant with a *p*-value of 0.001. X1 exhibited a minimal effect on Y, indicated by an *F*^2^ value of 0.061. The confidence interval (CI) extended from 0.062 to 0.270, indicating a significant relationship at the 95% confidence level. These findings suggest that robust strategic commitment and leadership patterns can harmonise policies and effectively integrate them into school governance and disaster management planning.

The analysis of X2 indicated a positive effect of 47.2% (0.472), which was statistically significant for Y, with a *p*-value of 0.000, falling below the 0.05 threshold. X2 exhibited a moderate effect, indicated by an *F*^2^ value of 0.289, with a CI ranging from 0.342 to 0.598, which does not encompass zero. The significant value produced is closely linked to the educational practices and routine implementations by stakeholders, particularly given the research area’s susceptibility to various disasters. Education, outreach and both vertical and horizontal relationships should continue to engage the community. X2 demonstrates that this relationship is significant at the 95% confidence level.

The analysis of X3 indicated a similarity to X1, maintaining significance with a *p*-value of 0.000. The effect size was weak, as indicated by an *F*^2^ value of 0.056, and a positive correlation of 11.5% (0.115) was observed. This positive correlation stems from community knowledge and local empowerment, leading to coordination, albeit on a limited scale. The influence of X3 exhibited a CI that approached zero, spanning from 0.051 to 0.180. This relationship was significant at the 95% confidence level.

The results of X4 indicated an insignificant outcome, as evidenced by the *p*-value (0.685) and a negligible effect size, reflected in the *F*^2^ value (0.001). This is additionally evidenced by a minimal positive correlation of 0.017, or 1.7%, alongside a CI that includes zero, spanning from −0.070 to 0.091. This is evident when a minimal and inconsequential influence is reflected in actual infrastructure practices. It affirms that physical factors alone do not entirely dictate the effectiveness of governance.

X5 demonstrates a positive effect of 27.7% (0.277), which is statistically significant with a *p*-value of 0.000, indicating it is below the 0.05 threshold. The influence of X5 is moderate, indicated by an *F*^2^ value of 0.184 and a CI ranging from 0.178 to 0. The relationship is statistically significant at the 95% confidence level. Disaster education and a culture of resilience play a significant role in governance, albeit with a lesser impact compared to governance and capacity.

Variable X4 is the sole construct that is statistically insignificant, as the confidence interval includes zero, and the *p*-value is above the established threshold. Additionally, constructs X1, X2, X3 and X5 exhibit positive and significant effects, as indicated by the confidence interval not encompassing zero and the *p*-value being below 0.05.

The appropriateness and assessment of the PLS-SEM test are discussed to determine the model’s predictive capability. The components comprise model measurement statements, such as *R*^2^, *Q*^2^, *F*^2^, PLS Predict and GoF (Hair et al. 2019; Sarstedt et al. 2019). *R*^2^ represents the coefficient of determination, indicating the proportion of variance in the construct that can be accounted for by the latent constructs within the model (Wetzels, Odekerken-Schröder & Van Oppen 2009). A higher *R*^2^ indicates a stronger explanatory power for the endogenous construct. The values are classified as 0.75 (substantial), 0.50 (moderate) or 0.25 (weak), contingent upon the context (Hair et al. 2017, 2019).

*Q*^2^ serves as an indicator of predictive relevance, reflecting the model’s capacity to forecast the indicator values of the endogenous construct (Hair et al. 2014, 2019). The analysis of *Q*^2^ via blindfolding, under the condition that *Q*^2^ > 0, demonstrates that the model possesses predictive relevance to the construct. Goodness-of-fit refers to the extent to which a model aligns with the data. Standardised root mean square residual serves as an index for assessing model fit, where a value below 0.08 is deemed fit or acceptable (Henseler & Sarstedt 2013).

The *R*^2^ value of 0.851 suggests that 85.1% of the variation in variable Y is accounted for by the latent constructs within the structural model. Additionally, only 14.6% of the variation is attributed to external factors not accounted for in the model. This is classified as possessing significant explanatory power. It is essential to situate it within a comprehensive structural evaluation framework that includes significance and path effects, predictive relevance and potential risks, ensuring that claims extend beyond *R*^2^ figures to align with current debates and best practices in PLS-SEM analysis.

The *Q*^2^ value demonstrates a significant strength of the observed variables, with a quantified value of 0.846. The robust *F*^2^ value has a significant influence on all measurement metrics within the research model. The provisions of Henseler and Sarstedt (2013) are valid, as all observed constructs exhibit large dominant effect sizes and meet the established requirements.

The analysis of 22 research indicators across 5 constructs and 20 components, utilising RMSE and MAE, indicated that the measurement items were lower than those produced by the linear regression model. The findings suggest that the proposed PLS model exhibits moderate predictive capability. The research model’s GoF value was 0.249, indicating a moderate fit. Figure 1 presents the research’s structural model.

**FIGURE 1:**
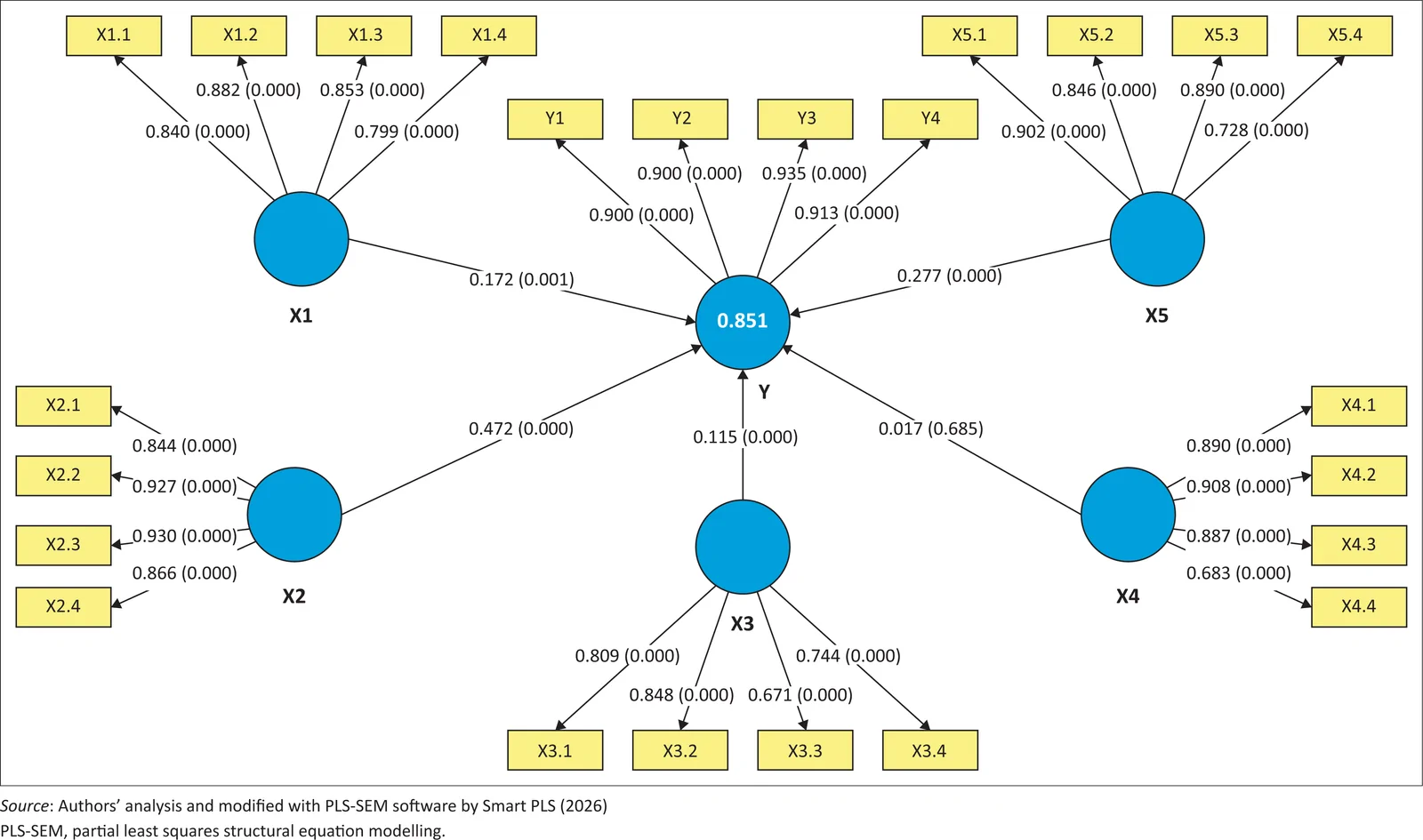
Integrated evaluation framework for disaster-smart school governance based on partial least squares structural equation modelling results.

The final DSSG framework should be read as an integrated evaluation tool for schools and policymakers. Risk Knowledge (X2) and Learning, Monitoring and Accountability (X5) serve as the core dimensions because they show the strongest associations with DSSG. Leadership and Strategic Commitment (X1) and Stakeholder Participation (X3) function as enabling dimensions that support implementation and legitimacy. Operational Preparedness/Resource Management (X4), although not statistically significant in this model, remains relevant as an operational component when embedded within risk-informed planning, accountability and continuous improvement. Therefore, the framework can be used to diagnose governance gaps, prioritise capacity-building interventions and guide school-level DRR planning.

Practically, the DSSG framework can be used by schools and local authorities as an evaluation tool to diagnose governance gaps and prioritise improvement. Schools may use the framework to assess whether risk knowledge is integrated into planning, whether preparedness activities are linked to accountability mechanisms, and whether monitoring results are used for continuous improvement. Local education authorities can also use the framework to identify schools requiring stronger technical support, resource allocation and policy supervision.

## Discussion

This study examined five governance dimensions within the DSSG framework in Padang, West Sumatra, Indonesia. The findings show that Risk Knowledge (X2) has the strongest positive association with DSSG, followed by Learning, Monitoring and Accountability (X5). Leadership and Strategic Commitment (X1) and Stakeholder Participation (X3) are also statistically significant, although their effect sizes are relatively small. In contrast, Operational Preparedness/Resource Management (X4) does not show a statistically significant association with DSSG. This pattern indicates that DSSG is not merely shaped by the existence of emergency plans, drills, facilities or resources, but by the extent to which these elements are embedded within risk knowledge, institutional learning, monitoring and accountability mechanisms.

The insignificant result for Operational Preparedness/Resource Management (X4) should not be interpreted as evidence that preparedness is unimportant. Rather, it indicates that operational preparedness may function as a necessary but insufficient condition for effective school disaster governance. Written emergency plans, drills, emergency facilities, resource allocation and continuity arrangements may become compliance-oriented or activity-based when they are not connected to risk assessment, institutional accountability and continuous evaluation. Therefore, the insignificant association of X4 suggests that operational instruments do not automatically strengthen governance unless they are integrated into a broader system of risk-informed decision-making and organisational learning.

This pattern informs our understanding of the functioning of disaster-smart schools within the evolving education system, which is moving towards an integrated evaluation framework. This structure of influence indicates that the effectiveness of school governance is influenced by multiple dimensions, particularly the consistent implementation of structural capacity and evaluation cycles (Fathani et al. 2023; Pearson & Pelling 2015; Putera et al. 2025).

Disaster-smart school governance cannot depend solely on individual behaviours; it necessitates a stable policy framework. These findings have sparked scientific debate, as the prevailing literature emphasises the importance of participatory leadership and a whole-of-society approach. Empirical findings demonstrate that structural capacity plays a significantly greater role than normative or symbolic factors.

This debate pertains to strategic commitment and leadership, which are influenced by institutional capacity, resources and political commitment. This phenomenon presents a challenge to implementation in Southeast Asian countries. Thailand integrated its post-2004 tsunami early warning system into its national coordination framework. Similarly, Japan has established a best practice by fostering a culture of preparedness from primary education, which includes regular evacuation drills and the development of earthquake-resistant infrastructure (Wirtz et al. 2014).

Empirical research across multiple nations underscores the significance of robust governance in disaster scenarios. Japan exemplifies community-based disaster governance practices that are underpinned by a national bureaucratic framework. Local governments possess the autonomy and capability to implement preparedness measures and establish disaster-resilient infrastructure (Ryan et al. 2020; Shaw et al. 2016).

Research in the Philippines indicates that decentralisation may undermine coordination unless supported by sufficient institutional capacity (Bankoff & Hilhorst 2009; Inan et al. 2018). This illustrates that governance involves not only institutional design, but also the implementation and practical capacity. The findings offer a significant theoretical contribution by differentiating between normative participation, as reflected in policy documents, and effective participation, which impacts resource allocation, strategic decisions and the implementation of enacted policies.

The relations between exogenous and endogenous constructs highlight that an integrated evaluation framework for DSSG must prioritise institutional capacity (X2) and evaluation mechanisms (X5), as both are critical for the effective implementation of DSSG. Leadership and coordination, while important, should be regarded as supporting factors that serve as enablers rather than the primary key associated governance dimension of success. In contrast, variable X4, typically regarded as significant in the normative literature on operational preparedness/resource management (X4), lacks empirical relevance. It requires reformulation to more precisely reflect the quality of social influence, rather than merely the frequency of involvement.

The relatively weak effect of Stakeholder Participation (X3) also requires further interpretation. Disaster governance literature commonly emphasises participation as a central principle of legitimacy, inclusion and community-based risk reduction. However, the present findings suggest that participation at the school level may not yet operate as a decisive governance mechanism. One possible explanation is that participation is often limited to attendance, consultation or involvement in routine activities, rather than meaningful influence over risk priorities, resource allocation, emergency decision-making and evaluation. Thus, the finding does not reject the importance of participation, but qualifies it: participation contributes more substantially to DSSG when it is empowered, substantive and institutionally connected to decision-making processes.

This study corroborates previous research on the importance of structure, resources and institutional capacity in enhancing school resilience, while also prompting a discussion of existing literature that highlights leadership and civic engagement in public governance, particularly in the realm of DRR.

Our research suggests that effective school governance in disaster contexts cannot be fully understood through normative or symbolic frameworks alone; it requires a more detailed, process-oriented and structural analysis. The primary impacts of X2 and X5 reflect institutional robustness, appropriate resource distribution, reliable assessment systems and enduring feedback processes, as opposed to the often-idealised notions of leadership or participation found in global frameworks like the Sendai Framework and the CSSF.

These findings refine the debate between actor-centred and system-centred approaches in disaster governance. Actor-centred perspectives remain important because leadership, stakeholder engagement and operational preparedness/resource management help produce legitimate and responsive DRR. However, the results of this study indicate that actor engagement becomes more effective when supported by system-level conditions, including risk knowledge, monitoring routines, accountability channels and institutional learning. Thus, the study does not reject actor-centred governance; rather, it argues that leadership and participation need to be embedded within a system-centred governance architecture to generate sustained and measurable improvements in DSSG.

From this perspective, DSSG should be understood as a structured and iterative governance process rather than a collection of isolated preparedness activities. The stronger associations of Risk Knowledge (X2) and Learning, Monitoring and Accountability (X5) indicate that schools become more disaster-smart when they can transform risk information into institutional routines, feedback mechanisms and improvement cycles. This interpretation is consistent with risk governance and resilience scholarship, which stresses that adaptive capacity depends not only on the presence of actors or preparedness activities, but also on the quality of coordination, learning, accountability and institutional adaptation over time.

This research presents empirical evidence indicating that leadership and participation alone do not guarantee improved governance in the absence of robust structural and evaluative foundations. It advocates a reformulation of disaster evaluation frameworks to prioritise institutional quality and ongoing policy learning. This suggests that disaster resilience is not a fixed state but a dynamic capacity developed through ongoing governance processes, which can sustain resilience over time.

Finally, the interpretation of these findings should be situated within the cross-sectional design of the study. Although the PLS-SEM results provide evidence of significant structural associations among governance dimensions, they do not establish definitive causality or temporal ordering. Therefore, the terms ‘influence’ and ‘effect’ in this study are interpreted within the statistical logic of hypothesised structural relationships rather than as proof of structural association. Future longitudinal or mixed-methods research would be valuable to examine how DSSG evolves over time and how preparedness, participation, leadership and monitoring mechanisms interact during actual disaster events or repeated preparedness cycles.

## Conclusion

This study was motivated by the urgent need to strengthen DSSG in Indonesia, particularly in Padang, West Sumatra, a highly disaster-prone area exposed to earthquakes, tsunamis, floods, landslides and multi-hazard risks. By applying PLS-SEM to data from disaster-prone schools, this study examined how five governance dimensions are associated with the development of DSSG and the advancement of an integrated evaluation framework.

The findings indicate that Risk Knowledge (X2) is the strongest and most statistically significant dimension associated with DSSG, followed by Learning, Monitoring and Accountability (X5). Leadership and Strategic Commitment (X1), as well as Stakeholder Participation (X3), show significant but relatively small effects, suggesting that they function more as enabling conditions than as a dominant key associated governance dimension. In contrast, Operational Preparedness/Resource Management (X4) does not show a statistically significant association with DSSG. This finding indicates that written emergency plans, drills, emergency facilities, resource allocation and continuity arrangements do not automatically translate into stronger governance unless they are integrated into risk-informed planning, institutional accountability and continuous learning mechanisms.

Theoretically, this study contributes to disaster governance literature by demonstrating that DSSG requires more than actor commitment, participation or operational readiness. It requires an integrated governance architecture that connects risk knowledge, institutional routines, monitoring, feedback and accountability. The findings therefore refine actor-centred perspectives by showing that leadership and participation remain important, but their contribution becomes more meaningful when embedded within system-centred governance processes.

Practically, the study recommends that school and local education authorities strengthen risk knowledge systems, establish routine monitoring and evaluation mechanisms and integrate DRR into school improvement planning. Operational preparedness should not be treated merely as a checklist of facilities, drills or written plans, but as part of a broader governance cycle that supports decision-making, resource allocation, accountability and continuous improvement. Stakeholder participation should also be strengthened beyond formal involvement by ensuring that teachers, students, parents, communities and local authorities have meaningful roles in planning, implementation and evaluation.

This study has limitations. Its geographic scope is limited to Padang, West Sumatra, which may restrict the generalisability of the findings to other regions with different hazard profiles and governance capacities. In addition, the cross-sectional design supports the interpretation of associations among constructs but does not allow definitive causal inference. Future research should expand the study area, apply longitudinal or mixed-methods designs and develop a composite DSSG assessment tool that integrates institutional, social, pedagogical and infrastructural dimensions to provide a more holistic understanding of DSSG.
